# Predictors of unemployment status in people with relapsing multiple sclerosis: a single center experience

**DOI:** 10.1007/s10072-022-06029-4

**Published:** 2022-03-23

**Authors:** Tommaso Guerra, Antonella Pipoli, Rosa Gemma Viterbo, Nicola Manghisi, Damiano Paolicelli, Pietro Iaffaldano, Luigi Di Lorenzo

**Affiliations:** 1 Department of Basic Medical Sciences, Neurosciences and Sense Organs, University of Bari Aldo Moro, Bari, Italy; 2 Interdisciplinary Department of Medicine, University of Bari Aldo Moro, Bari, Italy

**Keywords:** Multiple sclerosis, Unemployment, Disabilty

## Abstract

**Background:**

Multiple sclerosis (MS) is the most common cause of nontraumatic chronic neurological disability affecting young adults during their crucial employment years.

**Objectives:**

To evaluate patients and disease related factors associated to unemployment in a cohort of relapsing–remitting (RR) MS patients.

**Methods:**

We included RRMS patients with a follow-up of at least 1 year. We collected data about years of school education and employment status. Patients underwent a neuropsychological evaluation using the Brief International Cognitive Assessment for Multiple Sclerosis (BICAMS). Demographic and clinical predictors of unemployment were assessed through a multivariable stepwise logistic regression model.

**Results:**

We evaluated 260 consecutive RRMS patients. Employed patients were less frequently female (68.4% vs 83.3%, *p* = 0.006), less disabled (median Expanded Disability Status Scale (EDSS) score: 2.0 (0–7.0) vs 2.5 (0–7.5), *p* < 0.001), with more years of school education (mean ± standard deviation (SD), years: 13.74 ± 0.30 vs 10.86 ± 3.47, *p* < 0.001). Female sex and a higher EDSS score resulted associated with a greater risk of unemployment (OR 3.510, 95% CI 1.654–7.448, *p* = 0.001; OR 1.366, 95% CI 1.074–1.737, *p* = 0.011, respectively), whereas a greater number of years of schooling and current disease-modifying therapy exposure resulted protective factors (OR 0.788, 95% CI 0.723–0.858, *p* < 0,001; OR 0.414, 95% CI 0.217–0.790, *p* = 0.008, respectively).

**Conclusions:**

Understanding work is pervasively influenced by consequences of MS, we confirmed the impact of demographic, physical, and cognitive factors on employment status in RRMS patients.

## Introduction

Multiple sclerosis (MS) can be considered the most common cause of nontraumatic chronic neurological disability affecting young adults: being diagnosed between the ages of 20 and 45, MS affects people during their crucial employment years [1, 2]. Young patients often experience difficulties in carrying out their specific work duties regularly and in progressing in their careers, leading to a retirement from work, sometimes too early in relation to their actual psychophysical conditions [3, 4].

A higher proportion of individuals with MS report reduced work participation, with a consequent lower income from paid work, and unemployment compared to the general population and other patient groups with chronic diseases [5]. Employment also plays a fundamental role in self-identity and in the development of social contacts [6].

In Italy, in 2017, only 48% of people with MS of working age (< 65 years) were employed, and of these, about 30% had to reduce their working hours, and 27% had changed their type of job. For 63% of these workers, the changes led to a reduction in their income of more than 30%. In addition, one in three patients said they had to leave work early because of MS, and one in two said that symptoms and manifestations of the disease prevented them from doing the job they would have liked or were qualified for [7].

Consequently, the socioeconomic burden of the disease makes it crucial to underline which factors are predictive of employment status in order to recognize patients at risk for impaired working abilities.

Previous investigations have already underlined the link between patients’ characteristics and unemployment in MS, such as female gender, age, less education years, greater physical disability, and a progressive course [4, 8, 9]. More recently, therapeutic strategies that prevent disability accrual should be considered as one more predictor of employment [10].

The characterization of individual work ability requires also the assessing of factors beyond physical disabilities, such as cognitive functions and neuropsychiatric symptoms (e.g., history of depressive episodes, anxiety symptoms fatigue) [11, 12]. These predictors showed differential effects when examined by age, especially for physical symptoms and considering the middle-aged cohort [13]. Large discrepancies in employment status within Europe demonstrate that country-related factors may also have an impact on working possibilities [14].

Health care providers should use all this information when making treatment decisions with the aim of preserving working abilities and adapting working conditions [12].

Here, we report the results of a single center cross-sectional study aimed to evaluate patients and disease related factors associated to unemployment in a cohort of relapsing–remitting (RR) MS patients.

## Materials and methods

### Data collection and study population

All the data about MS history, demographics, treatments, and regular follow-up of the MS patients followed at the Multiple Sclerosis Center of the University Hospital Policlinico of Bari are collected, according to the Italian MS Registry study [15], which was approved by the ethical committee at the “Azienda Ospedaliero–Universitaria–Policlinico of Bari.” Patients signed an informed consent that allows to use clinical data for research purposes.

The criteria for inclusion in the study were as follows: RRMS diagnosed according to the criteria of Mc Donald [16] with a follow-up at the MS center of at least 1 year. Patients enrolled were scheduled to undergo a follow-up visit between 2018 and 2019.

Patients attending the center for the first time and those diagnosed with other phenotypes of MS were excluded.

A cross-sectional study design was applied. All the information collected at the time of the study visit was used to evaluate the risk of unemployment.

The following data were extracted from the database: gender, date of birth, date of onset and date of diagnosis, Expanded Disability Status Scale (EDSS) score with complete information regarding functional scores (FS), total number or relapses, and disease-modifying therapies (DMTs) (start- and end-dates of all the administered DMTs, discontinuation and causes). We also collected data about years of school education and employment status. At the study visit, patients underwent a brief neuropsychological evaluation using the Brief International Cognitive Assessment for Multiple Sclerosis (BICAMS) [17]. The BICAMS included the California Verbal Learning Test® | Second Edition (CVLT®-II), a comprehensive, detailed assessment of verbal learning and memory deficits in older adolescents and adults; the Symbol Digit Modalities Test (SDMT), which has demonstrated remarkable sensitivity in detecting the presence of brain damage and changes in cognitive functioning over time; and with the Brief Visuospatial Memory Test—Revised (BVMT-R), an assessment tool to measure visuospatial learning.

### Statistical analysis

In descriptive analyses, the results were expressed as mean ± standard deviation (or as median and interquartile range [IQR]) for continuous variables and as absolute frequency and percentage for categorical variables. Comparisons between groups of interest were formally conducted with Student’s *T* test or the chi-square test, depending on the variable’s nature.

Demographic and clinical predictors of unemployment were assessed through a multivariable stepwise logistic regression model, considering as dependent variable the unemployment and as independent variables sex, age, disease duration, EDSS, DMT exposure (yes/no), CVLTII, SDMT, and BVMTR scores and years spent on formal school education. Risks were reported as odds ratio (OR) with 95% confidence interval (CI). The multivariable stepwise logistic regression model, using the same variables, has been performed also considering the male and female cohort separately. Data analyses were performed by SPSS 25.0. The *p* values < 0.05 were considered significant.

## Results

During the years 2018–2019, 260 consecutive RRMS patients fulfilling the inclusion criteria attended the periodic clinical assessment.

Of these, 195 were female (75%) and 65 males (25%). All patients had received at least one DMT during their previous history.

In our patient cohort, at the time of the analysis 146 were currently employed and 114 were currently not working. In the group of patients currently employed, 67 subjects carried out purely intellectual work and 82 subjects reported mainly manual mansion.

Clinical and demographic characteristics of patients divided stratified by employment status are shown in Table 1.

**Table 1:** Clinical and demographic characteristics of patients stratified by employment status

Variable	Unemployed (*n* = 114)	Employed (*n* = 146)	*p* value
Female sex, *n* (%)	95 (83.3)	100 (68.4)	0.006
Current age (mean ± SD), years	40.27 ± 1.28	40.93 ± 0.89	0.649
EDSS score, median (IQR)	2.5 (0–7.5)	2.0 (0–7.0)	< 0.001
Disease duration (mean ± SD), years	8.93 ± 0.68	8.86 ± 0.56	0.892
Years of school education (mean ± SD) years	10.86 ± 3.47	13.74 ± 0.30	< 0.001
Patients with impaired CVLTII test results, *n* (%)	48 (42.1)	44 (30.1)	0.045
Patients with impaired SDMT test results, *n* (%)	45 (42.9)	40 (27.4)	0.039
Patients with impaired BVMTR test results, *n* (%)	38 (33.3)	21 (26)	0.199
Patients in treatment with DMTs, *n* (%)	65 (57)	52 (35.61)	< 0.001
Age at diagnosis (mean ± SD), years	32.20 ± 1.14	33.31 ± 0.88	0.419

Employed patients were less frequently female (68.4% vs 83.3%, *p* = 0.006), less disabled (median (IQR) EDSS score: 2.0 (0–7.0) vs 2.5 (0–7.5), *p* < 0.001), with more years of schooling (mean ± SD, years: 13.74 ± 0.30 vs 10.86 ± 3.47, *p* < 0.001). At the time of the analysis, employed patients treated with a specific DMT were 52 (35.6%) vs 65 (57%) unemployed patients (*p* < 0.001).

No differences were found in terms of age at MS diagnosis and in terms of current age.

Employed patients have almost the same age at time of the analysis (mean ± SD, years: 40.93 ± 0.89 vs 40.27 ± 1.28, *p* = 0.649) and have almost the same disease duration (mean ± SD, years: 8.86 ± 0.56 vs 8.93 ± 0.68, *p* = 0.892) of the unemployed patients.

In the group of unemployed patients, there was also a greater number of subjects presenting impaired neuropsychological functions: 48 patients (42.1%) vs 44 patients (30.1%) obtained impaired CVLTII test results (*p* = 0.045), 45 (42.9%) vs 40 (27.4%) had impaired SDMT test results (*p* = 0.039), and 38 (33.3%) vs 21 (26%) patients had impaired BVMTR test results (*p* = 0.199), in comparison with employed patients.

Table 2 shows the demographic and clinical characteristic of the two groups of patients (employed/unemployed) stratified by gender.

**Table 2:** Baseline characteristics of unemployment and employed patients stratified by sex

	Unemployed patients (114)			Employed patients (146)			
Variable	Female (*n* = 95)	Male (*n* = 19)	*p* value	Female (*n* = 100)	Male (*n* = 46)	*p* value	
Age at the time of analysis (mean ± SD), years	42.50 ± 1.37	37.00 ± 3.62	0.278	40.13 ± 1.04	42.61 ± 1.68	0.321	
EDSS score, median (IQR)	2.5 (0–7.5)	3.0 (1.0–7.0)	0.401	2.0 (0–7.0)	2.0 (0–7.0)	0.033	
Disease duration (mean ± SD), years	8.72 ± 0.76	10.06 ± 1.36	0.177	8.35 ± 0.63	9.91 ± 1.14	0.412	
Years of school education (mean ± SD) years	10.70 ± 0.36	11.71 ± 1.00	0.330	14.07 ± 0.36	13.04 ± 0.54	0.170	
Patients with impaired CVLTII test results, *n* (%)	40 (42.1)	8 (42.1)	0.659	28 (28)	16 (34.8)	0.407	
Patients with impaired SDMT test results, *n* (%)	39 (41)	6 (31.5)	0.441	24 (24)	16 (34.8)	0.175	
Patients with impaired BVMTR test results, *n* (%)	32 (33.6)	6 (31.5)	0.859	24 (24)	14 (30.4)	0.410	
Patients in treatment with DMTs, *n* (%)	54 (56.8)	11 (57.9)	0.933	39 (39)	13 (28.3)	0.208	
Age at diagnosis (mean ± SD), years	32.80 ± 1.20	29.02 ± 3.31	0.174	33.03 ± 1.01	33.88 ± 1.74	0.911	

The multivariable stepwise logistic regression model performed to estimate the predictors of unemployment retained the following factors: sex, age, disease duration, EDSS, specific treatment with DMT (yes/no), CVLTII, SDMT, and BVMTR results and years of school education. More in details, female sex and a higher EDSS score resulted associated with a greater risk of unemployment (OR 3.510, 95% CI 1.654–7.448, *p* = 0.001; OR 1.366, 95% CI 1.074–1.737, *p* = 0.011, respectively), whereas a greater number of years of schooling and current DMT exposure resulted protective factors against unemployment (OR 0.788, 95% CI 0.723–0.858, *p* < 0,001; OR 0.414, 95% CI 0.217–0.790, *p* = 0.008, respectively) (Table 3).

**Table 3:** Multivariable stepwise logistic regression model performed on the entire cohort to estimate the predictors of unemployment

Variable	OR (95% CI)	*p* value
Female sex	3.510 (1.654–7.448)	< 0.001
Age at the time of analysis	0.972 (0.943–1.003)	0.078
Disease duration	1.018 (0.972–1.067)	0.540
Last EDSS score	1.366 (1.074–1.737)	0.011
Treatment with DMT	0.414 (0.217–0.790)	0.008
Impaired CVLTII test results, *n* (%)	1.328 (0.681–2.590)	0.405
Impaired SDMT test results, *n* (%)	1.387 (0.698–2.754)	0.350
Impaired BVMTR test results, *n* (%)	0.639 (0.314–1.298)	0.215
Years of school education	0.788 (0.723–0.858)	< 0.001

The multivariable stepwise logistic regression model has been performed also considering the male and female cohort separately. In the female cohort, a higher EDSS score resulted associated with a greater risk of unemployment (OR 1.475, 95% CI 1.092–1.993, *p* = 0.011) and a greater number of years of schooling resulted a protective factor of unemployment (OR 0.761, 95% CI 0.688–0.843, *p* < 0,001). In the male cohort, no statistically significant associations were found.

## Discussion

The present investigation examined the results of a single center cross-sectional study aimed to evaluate the factors associated to unemployment in RRMS patients. The framing of the factors associated with a higher risk of unemployment becomes crucial in the understanding of patients with MS, because work is characterized as an expression of the individual and is pervasively influenced by the consequences of the disease.

According to the American Occupational Therapy Association, work has to be considered not only employment, but an occupation that includes interests and work activities, job search and acquisition, job performance, retirement preparation and adaptation, voluntary participation (https://www.aota.org/).

Several studies have been performed to evaluate the employment situation of a young adult diagnosed with a MS [2, 9, 12, 18].

According to AIMS Barometer 2021, 88% of people with MS have had stable and/or continuous employment in their life: for men the value is 95%, while for women 85%, confirming the greater difficulty for women with MS to access stable work [19].

Accordingly, the majority of unemployed patients were women. Unemployed patients were also characterized by fewer years of schooling and a slightly higher EDSS score. In the group of unemployed patients, there was also a greater number of subjects presenting impaired scores on neuropsychological assessments.

The multivariable stepwise logistic regression model performed to estimate the predictors of unemployment revealed that higher educational attainment and treatment with DMT can play a protective factor associated with a reduced risk of unemployment. On the contrary, female sex and a higher EDSS score resulted associated with a greater risk of unemployment.

In line with other studies [4, 8, 9], in our cohort, the majority of unemployed patients were women, with fewer years of schooling, slightly higher EDSS score, and higher disability.

It is therefore likely that, already at the official onset of the disease, the lower female employment is not only due to the importance of its clinical manifestations but also to the greater difficulties that women of the Italian and international general population encounter, at all ages, compared to males in obtaining a regularly paid job. In fact, according to data from the International Labor Organization in Italy, in the age group between 18 and 65, female workers are 48.9% vs 67.1% of males [20].

On the contrary, while earlier studies have reported a greater risk of unemployment in males with MS [8, 21], other studies did not consider gender significantly associated with employment status [2, 22].

However, gender inequality must be framed by considering differences in local socioeconomical, cultural, and legislative factors, which are also reflected in the cohorts of previous studies considered. Our results could be seen as an encouragement for women to stay at work to prolong workability in MS.

In a recent report about demographic and clinical factors leading to unemployment in MS patients in Poland, multivariate analysis revealed that moderate level of neurological disability (EDSS > 3) (OR = 11.089, *p* < 0.01), more severe fatigue (FSS > 4), and less present self-motivation were independently associated with unemployment [23].

Kirk-Brown and colleagues [24] analyzed data of a prospective sample of 673 employed patients responding to three annual self-report surveys. At follow-up, 87% had disclosed their MS diagnosis, and 16% had become unemployed, and regression models predicting disclosure revealed a significant association with self-reported physical disability. A higher EDSS score is associated to reduced work capacity [25] and long-term work absence and higher unemployment rates [4, 26, 27].

Often, the person with MS faces difficulties in returning to work after a relapse or in keeping the job over time: clinical phenotypes characterized by high disease activity and a high number of relapses therefore have a greater impact on absenteeism from work.

The therapeutic scenario for RRMS has widely expanded during the past 20 years, and choosing the MS therapy has become increasingly complex, due to the difficulties in weighing the risk/benefit ratio of several different available disease DMT, essential to prevent long term disability accumulation [28]. The multivariable stepwise logistic regression model performed in our study showed that treatment with a DMT at the time of analysis was also associated with a lower risk of unemployment.

In a recent study including Australian MS Longitudinal Study collected data from participants on DMTs usage from 2010 to 2015, patient-reported data have been used to assess benefits of DMTs on employment outcomes [29].

Patients in treatment with DMTs, in this case in particular with fingolimod and natalizumab, reported significant increases in amount of work, work attendance, and work productivity, suggesting an important benefic effect on work life [29].

Cognitive impairment (CI) and fatigue are common features of multiple sclerosis (MS), with an estimated prevalence ranging approximately from 40 to 65% of patients [30, 31].

The most common affected cognitive domains in MS are information processing speed, abstract reasoning, executive functioning, sustained attention, and long-term memory. All these aspects are functional to the proper performance of the tasks required at the workplace.

O’Connor et al. [32] indicates that impaired memory and concentration are important factors conditioning employment. Another study [33] pointed out that fatigue, slowed processing speed, and impairments in memory are major factor in the risk unemployment. In fact, in our cohort, the group of unemployed patients was characterized by a greater number of subjects presenting impaired scores on neuropsychological assessments, even if this association was not confirmed in our multivariable logistic model.

Our results have identified schooling as a significant factor in the ability to remain in employment. Other studies have already highlighted the role of low-education level as a predictor of work loss and unemployment [4, 34].

Some limitations of this study need to be acknowledged. Although the literature in this regard emphasizes its importance, we do not include a formal testing of fatigue. Anyhow, at least indirectly we took in consideration this aspect, because it is well known that higher EDSS scores and the presence of impaired cognitive functions are associated with the presence of fatigue. A longer follow-up in a larger population is needed to confirm the factors associated with unemployment risk we found in this study, hold-up in the long-term.

All the above considerations, highlighting the objective difficulties of MS patients in the world of work, constitute an important proof of the importance of an early diagnosis, in order to decide the correct therapy and to reduce the impact of the disease on physical and cognitive aspects.

In addition, in a forthcoming study, we would like to evaluate in more detail the characteristics of MSRR patients, especially females, who maintain employment compared to those who abandon it, in order to better understand whether work can be a health- promoting tool for these individuals and to be able to provide them with more adequate support for maintaining employment.
